# Genome-wide identification and molecular characterization of the *AP2/ERF* superfamily members in sand pear (*Pyrus pyrifolia*)

**DOI:** 10.1186/s12864-022-09104-4

**Published:** 2023-01-19

**Authors:** Yue Xu, Xiaona Li, Xiong Yang, Misganaw Wassie, Haiyan Shi

**Affiliations:** 1 College of Horticulture, Hebei Agricultural University, Baoding, 071001 Hebei China; 2 Wuhan Botanical Garden, Chinese Academy of Sciences, Wuhan, 430074 Hubei China

**Keywords:** *Pyrus pyrifolia*, *AP2/ERF* genes, Ripening and senescence, Molecular characterization, Expression introduction

## Abstract

**Background:**

‘Whangkeumbae’ (*Pyrus pyrifolia*) is a typical climacteric fruit variety of sand pear with excellent taste. However, the rapid postharvest ethylene production limits the shelf life of ‘Whangkeumbae’ fruit. AP2/ERF superfamily is a large family of transcription factors involved in plant growth and development, including fruit ripening and senescence through the ethylene signaling pathway. The numbers and functions of AP2/ERF superfamily members in sand pear remain largely unknown.

**Results:**

In this study, a total of 234 AP2/ERF family members were identified through the transcriptome of *Pyrus pyrifolia* ‘Whangkeumbae’ (17 genes) and *Pyrus pyrifolia* genome (223 genes) analyses. Six genes (Accession: EVM0023062.1, EVM0034833.1, EVM0027049.1, EVM0034047.1, EVM0028755.1, EVM0015862.1) identified via genome analysis shared 100% identity with *PpERF14-L*, *PpERF5-L*, *PpERF3a*, *PpERF3*, *PpERF017* and *PpERF098,* respectively, which were identified from transcriptome sequencing. Further, the AP2/ERF superfamily members were divided into AP2, ERF, and RAV subfamilies, each comprising 38, 188, and 8 members, respectively. Tissue-specific expression analysis showed that *PpERF061*, *PpERF113, PpERF51L-B*, *PpERF5-L,* and *PpERF017* were predominantly expressed in fruits than in other tissues. Additionally, *PpERF5-L* and *PpERF017* showed higher expressions at the early stage of fruit development. While, *PpERF51B-L* exhibited higher expression during the fruit ripening stage. Besides, *PpERF061* and *PpERF113* had pronounced expressions during fruit senescence.

**Conclusion:**

These results indicate that *PpERF061*, *PpERF113*, *PpERF51L-B*, *PpERF5-L,* and *PpERF017* could play crucial roles in sand pear fruit development, ripening, and senescence. Overall, this study provides valuable information for further functional analysis of the *AP2/ERF* genes during fruit ripening and senescence in sand pear.

**Supplementary Information:**

The online version contains supplementary material available at 10.1186/s12864-022-09104-4.

## Introduction

‘Whangkeumbae’ is a typical climacteric fruit variety of sand pear (*Pyrus pyrifolia*), known for its smooth surface and good flavor. After ripening, the fruit experience various physiological changes, such as increased sugar and ethylene content as well as change in fruit color and firmness [1]. However, the rapid postharvest ethylene production reduces the shelf life of ‘Whangkeumbae’ fruit, thus limiting the industrial production of Whangkeumbae’. The phytohormone ethylene plays a crucial role in regulating fruit ripening and senescence [2].

The biosynthesis of ethylene consists of two sequential steps. Initially, 1-aminocypropane-1-carboxylic acid (ACC) synthase (ACS) converts the ethylene precursor S-adenosine methionine (SAM) into ACC, and later ACC oxidase (ACO) oxidizes ACC into ethylene [3]. The ethylene signaling pathway is activated by the ethylene insensitive 3 (EIN3)/EIN3-LIKE (EIN3/EIL) transcription factors (TFs), which in turn activates ethylene response factor (ERF) TFs that regulate the expression of ethylene-responsive genes [4]. ERF TFs belong to the APETALA2 (AP2) /ERF superfamily [5].

*AP2/ERF* superfamily is a large gene family of transcription factors involved in plant growth, development, and biotic and abiotic stress responses [6–10]. Given their crucial roles, the *AP2/ERF* superfamily members have been identified from various plant species and characterized during fruit ripening and senescence. In tomato, *LeERF1* gene aggravated ripening and softening in postharvest fruit [11]. *SlERF12* negatively modulates tomato fruit ripening by inhibiting the expression of fruit ripening genes through interaction with the co-repressor TOPLESS protein and the histone deacetylases [12]. Similarly, *MdERF4* interacted with TOPLESS corepressor 4 and recruited histone deacetylase (*MdHDA19*) suppress apple fruit ripening by inhibiting the acetylation of ripening-related genes [7, 13]. In ‘Zaosu’ pear, an ethylene response factor, *ERF22* was proven to promote anthocyanin biosynthesis [14]. In peach, *PpeERF2*, *PpeERF3*, Prupe.2G289500 and Prupe.1G037900 could regulate fruit ripening [15–17].

AP2/ERF superfamily members contain one or two well-conserved AP2 DNA binding domains comprising 60 to 70 amino acids (aa) [18]. Based on the number and variety of conserved domains, AP2/ERF superfamily is divided into AP2, ERF, and RAV subfamilies [19]. The AP2 subfamily consists of proteins containing two AP2 domains, while the ERF subfamily members contain a single AP2 domain. The RAV subfamily members contain one AP2 domain and an extra B3 domain [20–22]. Several members of the AP2/ERF superfamily have been identified from various plant species, including white pear [20], apple [18], peach [23], grape [24], kiwifruit [25], Arabidopsis [26], soybean [27], rice [5] and ginger [28]. However, information about the AP2/ERF superfamily members in sand pear is limited. Transcriptome [29] and genome-wide [30] sequencing of *Pyrus pyrifolia* provides an opportunity to identify AP2/ERF superfamily in sand pear.

In this study, genome-wide analysis was performed to identify members of the AP2/ERF family in sand pear based on the transcriptome and genome sequences of *Pyrus pyrifolia*. We identified 17 and 223 members of the AP2/ERF from the transcriptome and genome data, respectively. Interestingly, six *PpERF* genes identified from transcriptome data were identical to those identified from the genome data. After removing redundant genes, 234 unique AP2/ERF superfamily members were identified in sand pear. The three subfamilies of AP2/ERF, including AP2, ERF and RAV, comprised 38, 188, and 8 members, respectively. Furthermore, we analyzed the evolutionary relationships, chromosomal distribution, physicochemical properties, conserved motifs, protein domains, gene structures, and cis-elements of the 17 AP2/ERF members identified from transcriptome data. This study provides essential information for understanding of the function and evolution of the *AP2/ERF* gene family in sand pear.

## Results

### Identification and phylogenetic analysis of *AP2/ERF* genes in sand pear

A total of 223 *AP2/ERF* superfamily members were identified by combining of Hidden Markov Model (HMM) and BLAST search in the *Pyrus pyrifolia* genome. In our previous study, 17 proteins encoding the AP2/ERF domains were identified via transcriptome sequencing of ‘Whangkeumbae’ (*Pyrus pyrifolia*) [29]. Interestingly, six genes identified from the genome (Accession:EVM0023062.1, EVM0034833.1，EVM0027049.1，EVM0034047.1，EVM0028755.1，EVM0015862.1) shared 100% similarity with *PpERF14-L*，*PpERF5-L*，*PpERF3a*，*PpERF3*，*PpERF017* and *PpERF098,* respectively, which were identified from transcriptome analysis. Therefore, after removing redundant genes, 234 unique members of the *AP2/ERF* superfamily were identified in *Pyrus Pyrifolia*. The conserved domains analysis showed that the 234 members could be classified into the three subfamilies, including 38 AP2 (two AP2/ERF domains), 188 ERF (one AP2/ERF domain), and 8 RAV (one AP2/ERF and an extra B3 domain).

**Fig. 1:**
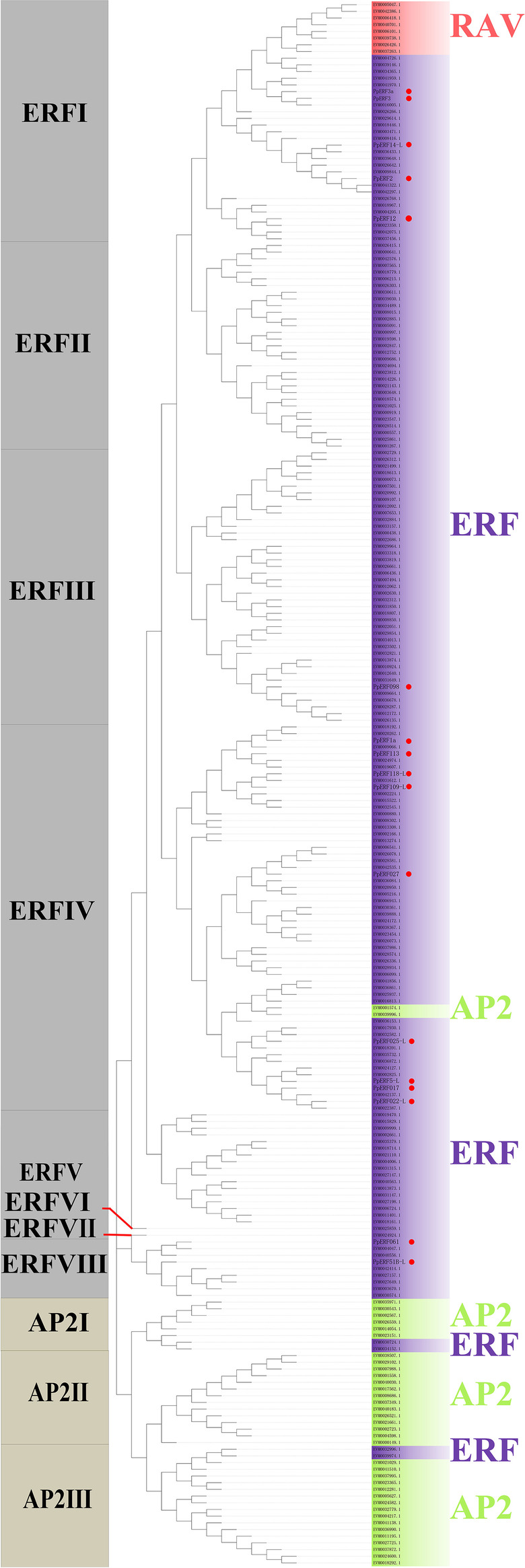
Phylogenetic tree and classification of 234 *AP2/ERF* gene family members in the *Pyrus pyrifolia* genome. The amino acid sequences of the 234 PpERFs proteins were aligned using ClustalW method and phylogenetic tree was constructed based on bootstrap analysis of 1000 replicates using the Neighbor-Joining (NJ) method

Further, phylogenetic analysis showed that the AP2/ERF members were divided into 11 distinct branches (Fig. 1). Among them, the ERF subfamily comprised 8 branches (I-VIII). The AP2 subfamily had three distinct groups (I-III). All the 17 genes identified from transcriptome analysis contained a single AP2/ERF domain and belonged to the ERF subfamily.

### Chromosomal localization and physicochemical properties of PpERFs

**Fig. 2:**
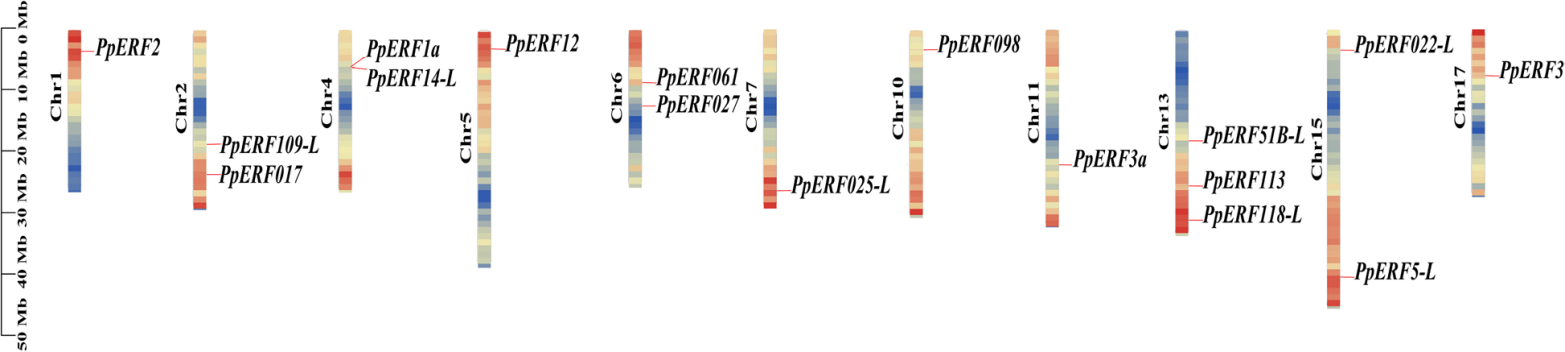
Schematic representations of the chromosomal distributions of the 17 PpERFs

The chromosomal localization analysis revealed that the 17 *AP2/ERF* genes were randomly distributed on the chromosome (Chr) of *Pyrus pyrifolia (*Fig. 2). *PpERF2*, *PpERF12*, *PpERF025-L*, *PpERF098*, *PpERF3a* and *PpERF3* were located on Chr 1, 5, 7, 10, 11 and 17, respectively. *PpERF109-L* and *PpERF017* were located on Chr 2; P*pERF1a* and *PpERF14-L* on Chr 4; *PpERF061* and *PpERF027* on Chr 6; *PpERF022-L* and *PpERF5-L* on Chr 15. The final three *AP2/ERF* genes, *PpERF51B-L*, *PpERF113* and *PpERF118-L* were found on Chr13.

We further investigated the physicochemical properties of the 17 PpERFs (Table 1). We observed noticeable variations in the number of amino acids and physicochemical properties among PpERF proteins. The length of PpERF proteins ranged from 149 aa (PpERF098) to 327 aa (PpERF061/PpERF14-L). The molecular weight (MW) ranged from 16.76 k Dalton (kDa) (PpERF098) to 36.74 kDa (PpERF14-L) and the isoelectric point (pI) varied from 4.75 (PpERF017) to 9.51 (PpERF12). The pI values of 11 PpERFs were weakly acidic, and that of the 6 PpERFs were alkaline, indicating that most of the AP2/ERF members of sand pear are rich in acidic amino acids. Moreover, the 17 PpERFs were found to be localized in the nucleus or cytoplasm. Further interaction prediction analysis suggested that PpERFs could interact with transcription factors or proteins.

**Table 1:** The characterizations of 17 PpERFs in pear

Protein name	Size (aa)	MW (Da)	pI	Chromosome position	Subcellular localization	Interaction
**PpERF51B-L**	229	25,649.36	5.10	Chr13	Nucleus	EIL3
**PpERF12**	178	18,828.17	9.51	Chr5	Nucleus	BOI
**PpERF109-L**	313	34,623.69	7.75	Chr2	Nucleus	UBR1
**PpERF027**	185	20,071.65	5.37	Chr6	Nucleus	NOP2
**PpERF061**	327	35,631.76	6.85	Chr6	Nucleus	NAC14
**PpERF118-L**	321	35,846.56	4.76	Chr13	Nucleus	ANK1
**PpERF025-L**	236	25,003.65	4.91	Chr7	Nucleus	NOP2
**PpERF14-L**	327	36,743.26	6.19	Chr4	Nucleus	MMK1
**PpERF5-L**	160	17,890.96	9.19	Chr15	Nucleus	NOL
**PpERF3a**	230	24,942.85	8.77	Chr11	Nucleus	HDA19
**PpERF1a**	279	30,194.88	9.11	Chr4	Nucleus	CSC1
**PpERF113**	250	27,706.34	6.92	Chr13	Nucleus	NAC72
**PpERF022-L**	201	22,334.04	5.28	Chr15	Nucleus	CLUH
**PpERF3**	227	24,689.61	7.04	Chr17	Nucleus	BHLH51
**PpERF017**	234	25,918.40	4.75	Chr2	Nucleus	DRMH3
**PpERF2**	246	27,884.18	5.92	Chr1	Nucleus	BEL1
**PpERF098**	149	16,761.59	5.76	Chr10	Nucleus	HSP90

*MW* molecular weight, *pI* isoelectric point, *aa* amino acids, *Da* Dalton

**Phylogenetics, conserved motifs, domains and gene structures of the 17 PpERFs.**

**Fig. 3:**
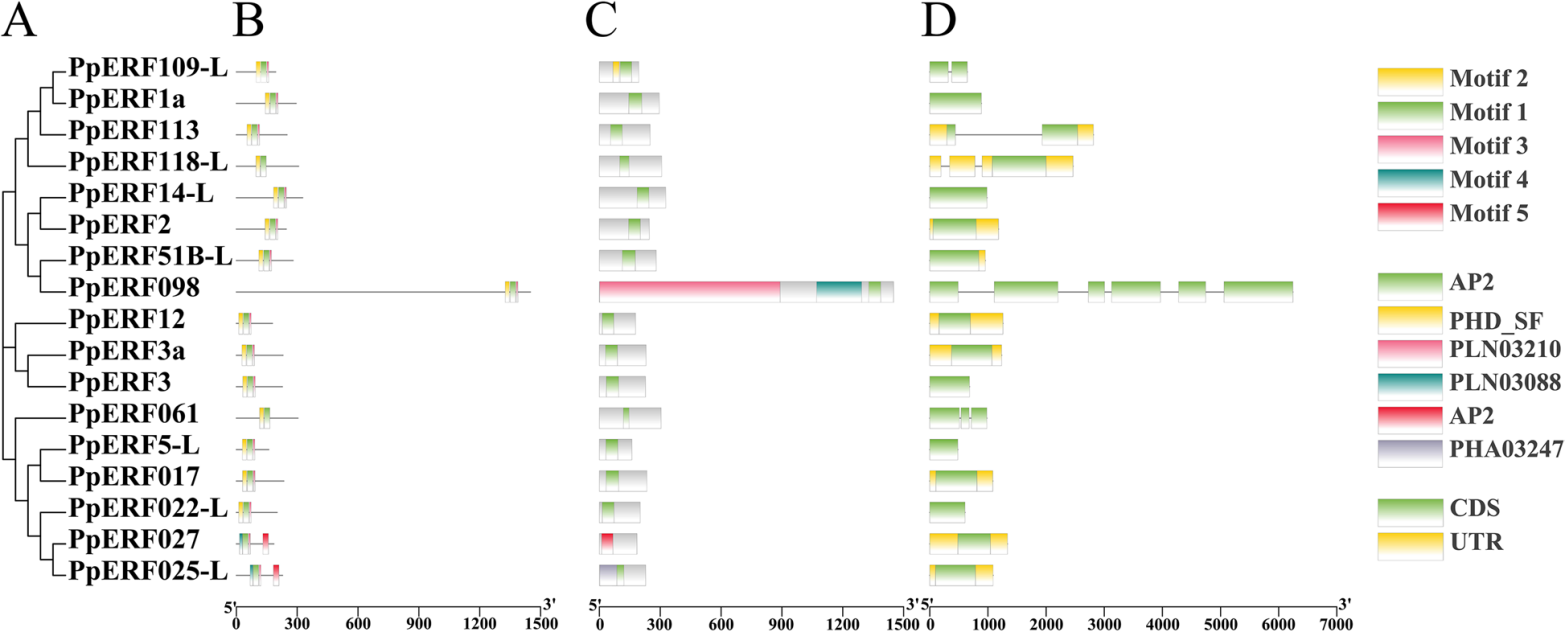
Evolutionary relationships, conserved protein motifs, domains and gene structures of the 17 PpERFs. **A** The phylogenetic tree was constructed based on protein sequences using the NJ method by MEGA7.0. **B**-**D** Motif compositions, domains and Exon/intron structures of the 17 PpERFs

To understand the relationship of the PpERFs, phylogenetic tree was constructed based on the protein sequences of the 17 AP2/ERF members (Fig. 3 A). They were divided into three clades: I, II, and III, each comprising 8, 3, and 6 PpERF members, respectively.

Motif analysis showed five conserved motifs across the PpERFs (Fig. 3 B). All 17 PpERFs contain a highly conserved motif 1. Additionally, all PpERFs, except PpERF12, PpERF027, PpERF025-l, and PpERF022-L had motif 2. Motif 1 and motif 2 were closely related to the function of the AP2 domain. Motif 3 was found in 15 PpERFs, but not in *PpERF061* and PpERF118-L. Motif 4 was found only in PpERF027, PpERF025-L, and PpERF022-L. Among the 17 PpERFs, only PpERF12, PpERF027 and PpERF025-L contained motif 5. Motif logos and site numbers of each motif are shown in Additional file 1 (Fig. S1). Furthermore, conserved domains were analyzed for the protein sequence of the 17 PpERFs. All PpERFs contained the AP2 domain and belonged to the ERF subfamily, consistent with the above evolutionary analysis (Fig. 3 C). Besides, PpERF109-L and PpERF025-L had an extra plant homeodomain (PHD) zinc finger domain and PHA03247 domain, respectively.

The predicted gene structure results showed that *PpERF1a*, *PpERF14-L*, *PpERF3*, *PpERF5-L,* and *PpERF022-L* had one exon. While *PpERF2*, *PpERF51B-L*, *PpERF12*, *PpERF3a*, *PpERF017*, *PpERF027,* and *PpERF025-L* had one exon and one/two UTR regions. Only *PpERF109-L*, *PpERF113*, *PpERF118-L*, *PpERF098,* and *PpERF061* found to have introns (Fig. 3 D).

**Cis-element analysis of 17** ***PpERF*** **gene promoters.**

**Fig. 4:**
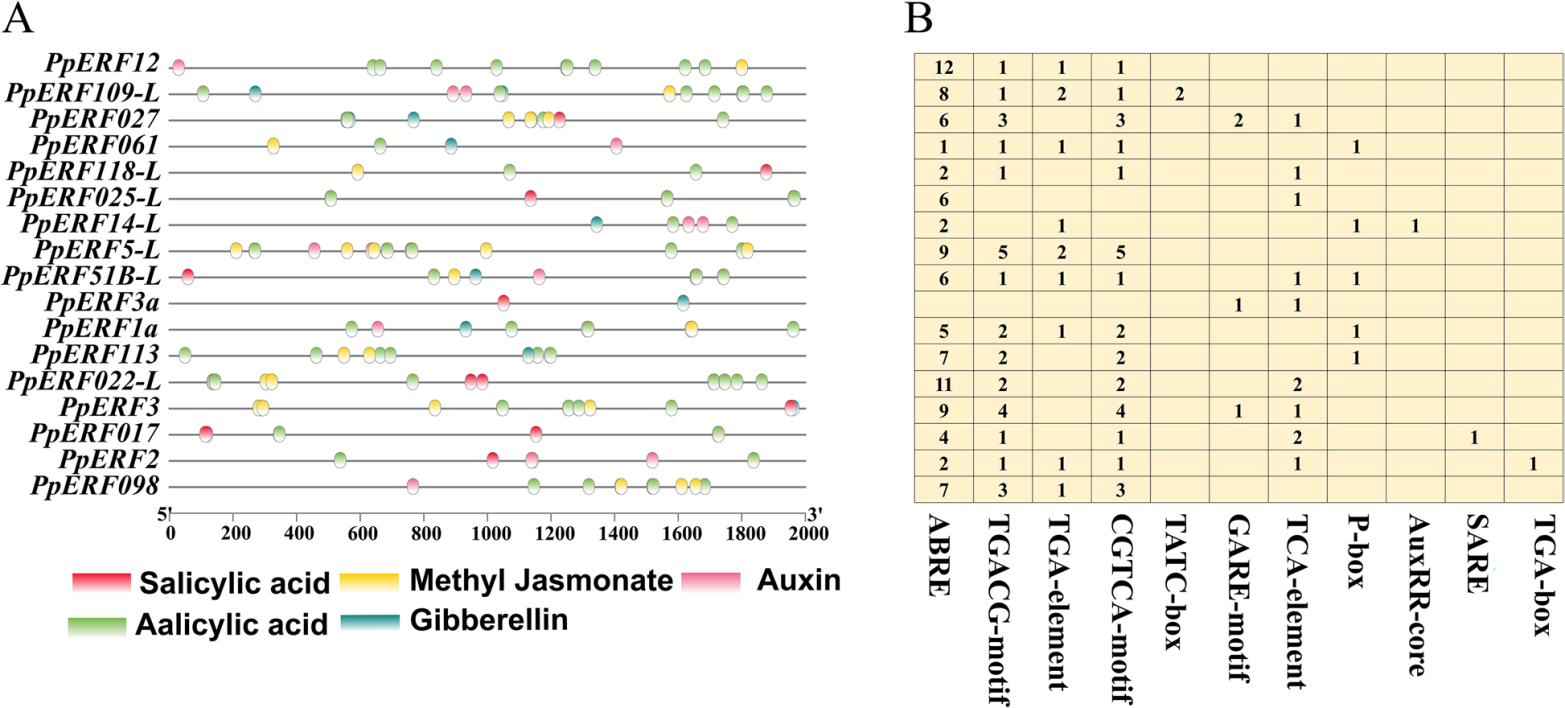
Cis-acting elements identified in the promoter regions of the 17 *PpERF* genes in *Pyrus pyrifolia*. **A** The information of species and localization. **B** The information of quantity

To identify the cis-elements, we analyzed the 2000 bp upstream sequences from the start codons of the 17 *PpERF* genes. We identified hormone-responsive elements in the putative promoter regions, including abscisic acid response, auxin response, gibberellin response, Methyl Jasmonate (MeJA) response, and salicylic acid response. The types and locations of these elements are shown in Fig. 4 A, and the number of elements is displayed in Fig. 4 B. The 17 *AP2/ERF* genes contained five hormone responsive-related motifs, comprised of 11 cis-elements.

All *PpERF* genes, except *PpERF3a* contained abscisic acid-responsive elements. *PpERF12*, *PpERF109-L*, *PpERF027*, *PpERF061*, *PpERF118-L*, *PpERF025-L*, *PpERF14-L*, *PpERF5-L*, *PpERF51B-L*, *PpERF3a*, *PpERF1a*, *PpERF113*, *PpERF022-L*, *PpERF3*, *PpERF017*, *PpERF2* and *PpERF098* include the MeJA elements. *PpERF12*, *PpERF109-L*, *PpERF061*, *PpERF14-L*, *PpERF5-L*, *PpERF51B-L*, *PpERF1a*, *PpERF2* and *PpERF098* genes had auxin response elements. We identified gibberellin-responsive elements in the promoter of *PpERF109-L*, *PpERF027*, *PpERF14-L*, *PpERF51B-L*, *PpERF3a*, *PpERF1a*, *PpERF113*, and *PpERF3*. *PpERF027*, *PpERF118-L*, *PpERF025-L*, *PpERF51B-L*, *PpERF3a*, *PpERF022-L*, *PpERF3*, *PpERF017*, and *PpERF2* genes contain salicylic acid-responsive elements in their promoter. These results suggest that hormonal-responsive elements may directly determine the regulatory role of the *AP2/ERF* genes family in sand pear fruit ripening and senescence.

### The expressions of *PpERFs* are regulated during fruit ripening and senescence

**Fig. 5:**
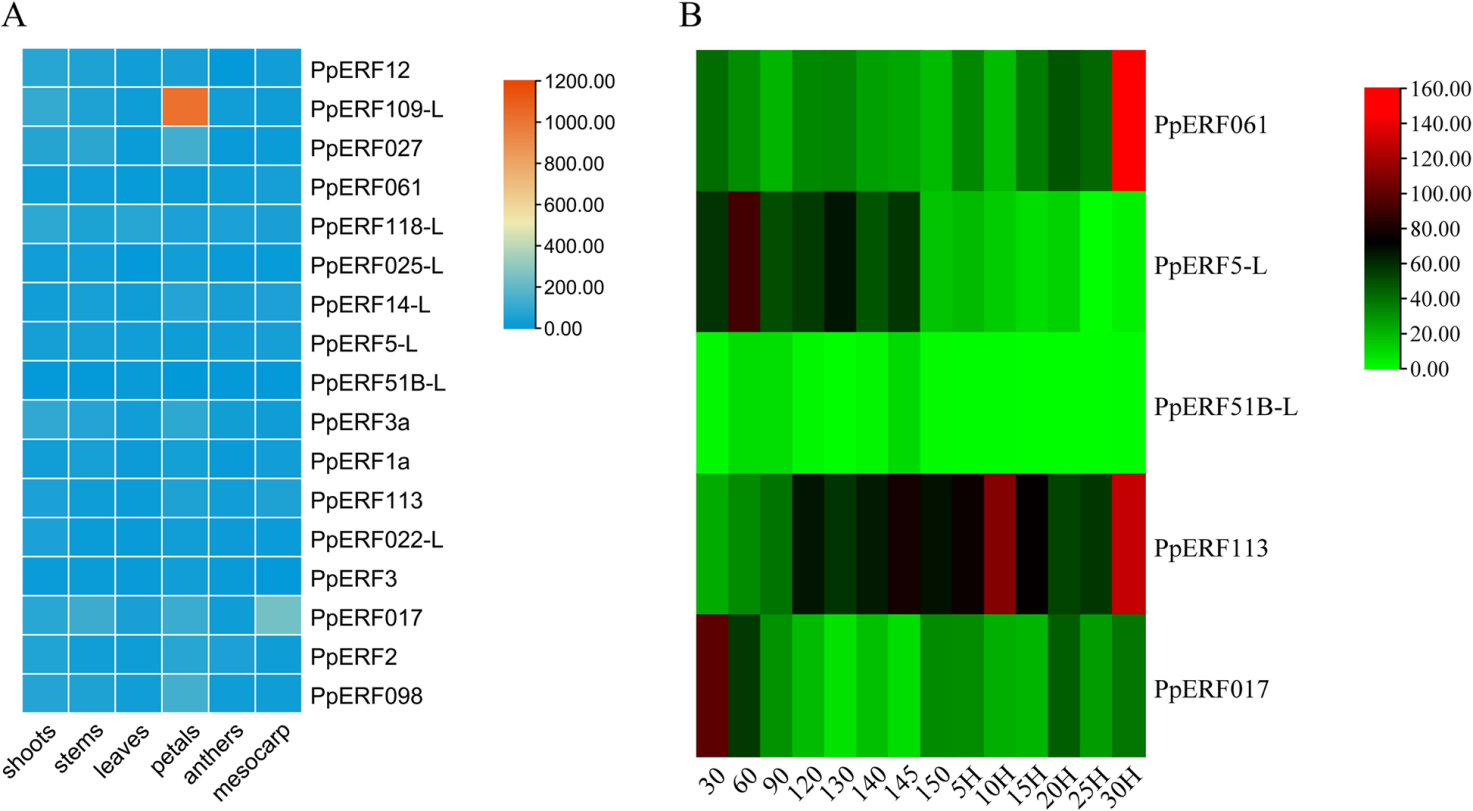
Hierarchical clustering analysis of the expression of *PpERFs* in ‘Whangkeumbae’ (*Pyrus pyrifolia*) tissues (**A**) and different fruit developmental stages (**B**)

**Fig. 6:**
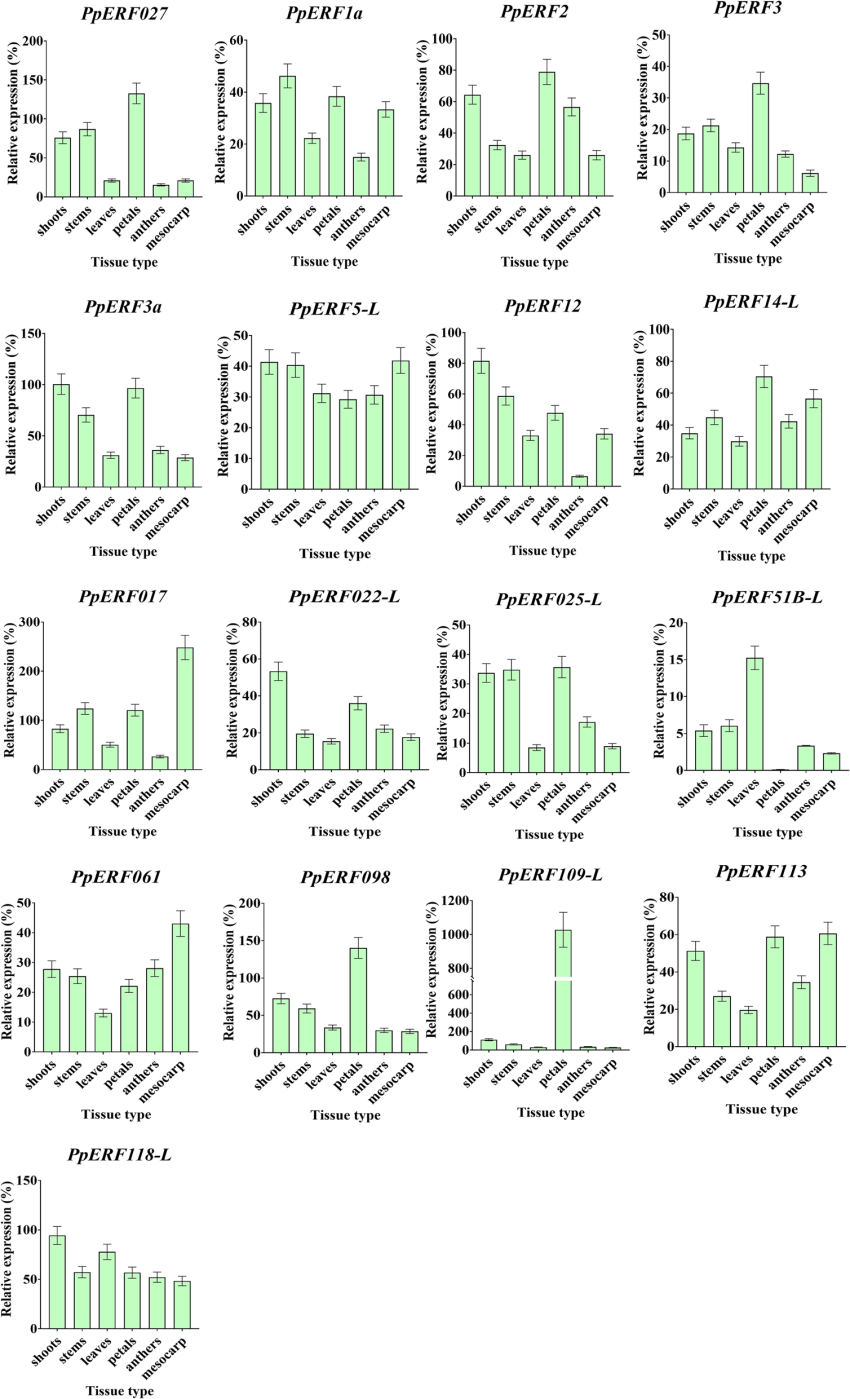
The qRT-PCR expression analysis of *PpERF027*, *PpERF1a*, *PpERF2*, *PpERF3*, *PpERF3a*, *PpERF5-L*, *PpERF027*, *PpERF12*, *PpERF14-L*, *PpERF017*, *PpERF022-L*, *PpERF025-L*, *PpERF51B-L*, *PpERF061*, *PpERF098*, *PpERF109-L*, *PpERF113* and *PpERF118-L* in ‘Whangkeumbae’ (*Pyrus pyrifolia*) tissues

Furthermore, we determined the expression patterns of the 17 differentially expressed genes (DEGs) in different tissues. The red color indicates strong expression, while blue color represents weak expression (Fig. 5 A). Moreover, the tissue-specific expression analysis showed that the 17 *PpERF* genes were expressed in flesh tissue, among which *PpERF061*, *PpERF11PpERF51L-B*, *PpERF5-L,* and *PpERF017* were significantly expressed. Notably, *PpERF109-L* was highly expressed in the petal (Fig. 6).

**Fig. 7:**
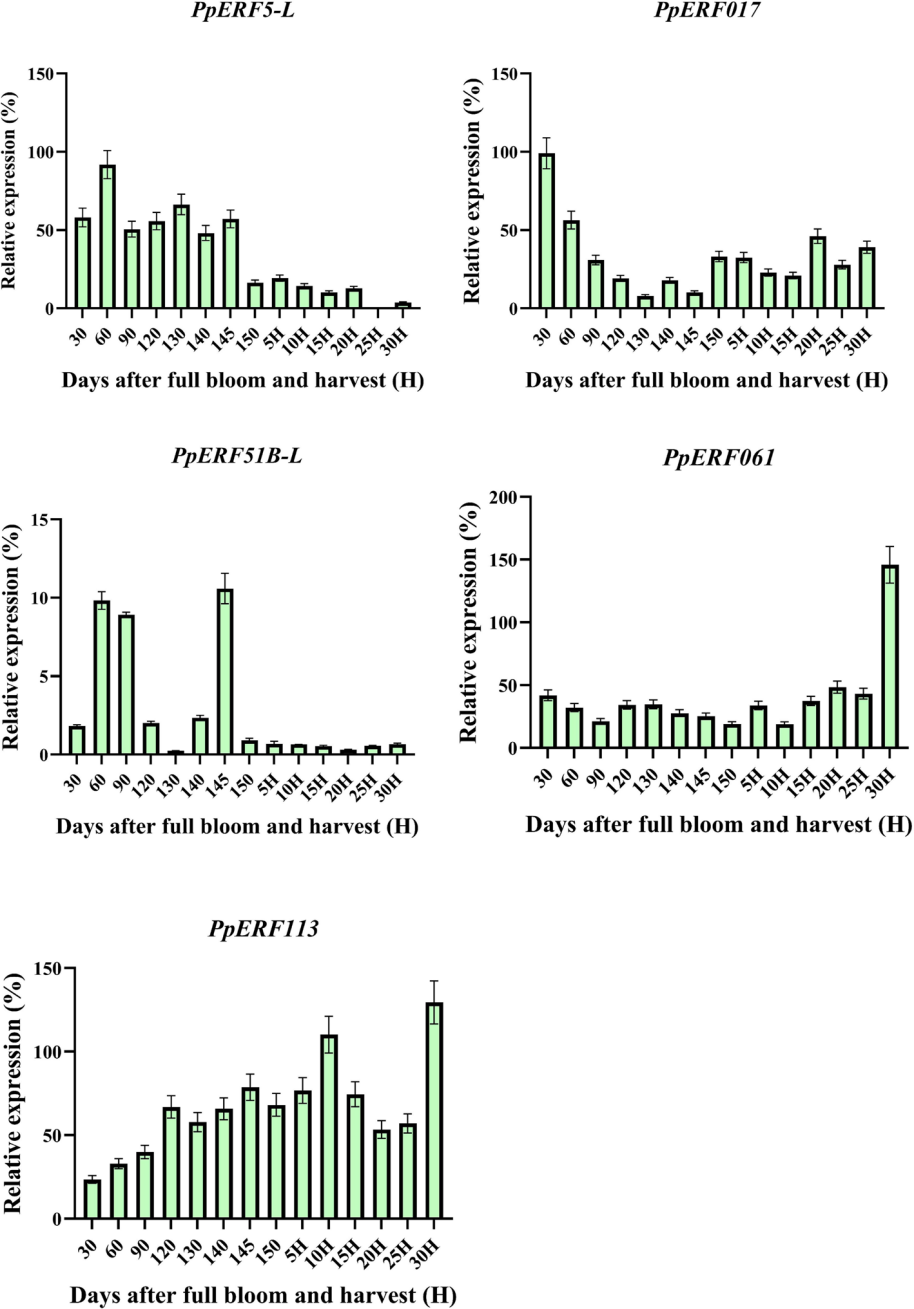
The qRT-PCR expression analysis of *PpERF5-L*, *PpERF017*, *PpERF51B-L*, *PpERF061*, *PpERF113* during fruit development, ripening, and senescence. The values are given as mean ± SD from three independent experiments

Five genes that showed differential expression in the flesh were selected for subsequent analysis. As shown in the heat map, the green color represents weak expression, while the red color indicates strong expression (Fig. 5 B). Cluster analysis showed that the expression levels of *PpERF061* and *PpERF113* were higher at 30 days after harvest (DAH) (Fig. 7). In contrast, the expression levels of *PpERF5-L* and *PpERF017* were significantly higher at the early stage of fruit development, while *PpERF51B-L* was mainly expressed during fruit ripening (Fig. 7). These results suggest that *PpERF061* and *PpERF113* genes may be involved in fruit senescence, while *PpERF5-L* and *PpERF017* genes may play a crucial role in fruit development. Additionally, *PpERF51B-L* may specifically regulate fruit ripening.

## Discussion

The *AP2/ERF* superfamily members have been identified from various plant species and functionally characterized during fruit ripening and senescence. The number of genes in the AP2/ERF superfamily are diverse among plant species (Additional file 2: Table S1). For instance, the *Pyrus bretschneideri* genome contains 191 *AP2/ERF* superfamily members, among which 101 belonged to the *ERF* subfamily [20]. There are 259 *AP2/ERF* members in *Malus domestica*, including 195 *ERF* genes [18]. In *Prunus persica*, there are 131 *AP2/ERF* superfamily members, including 104 *ERF* genes [23]. There are 122 *ERF* genes in *Vitis vinifera* [24] and 119 *ERF* genes in *Actinidia eriantha* [25]. In *Arabidopsis thaliana*, *AP2/ERF* superfamily contains 147 members, including 122 *ERF* genes [26]. There are 148 *AP2/ERF* genes in *Glycine max* [27], 180 *AP2/ERF* genes in *Oryza sativa* [5] and 163 *AP2/ERF* genes *Zingiber officinale* [28]. In this study, a total of 223 *AP2/ERF* superfamily members were identified in the *Pyrus Pyrifolia* ‘Cuiguan’ v1.0 genome. In our previous study, 17 *AP2/ERF* genes were identified through transcriptome analysis, and six genes (*PpERF14-L*，*PpERF5-L*，*PpERF3a*，*PpERF3*，*PpERF017* and *PpERF098*) were also identified through genome-wide analysis in the present study. After removing redundant members, 234 unique *AP2/ERF* gene family members were identified in sand pear. The number of genes in the *RAV* subfamily are highly conserved among different plant species. Here, we found large number of *AP2/ERF* superfamily members in sand pear, which could be due to the *AP2* and *ERF* members. Phylogenetic analysis revealed that the 234 PpERFs were divided into three different subfamilies (ERF, AP2, and RAV), in agreement with other plant species [5, 18]. In sand pear, ERF was the largest subfamily with 188 genes, including the 17 PpERFs. These results agreed with the distribution of *AP2/ERF* genes reported in other plant species [19]. Members of the ERF subfamily are also known as the ethylene-response element binding protein family [31].

Furthermore, various bioinformatics analyses were conducted for the 17 PpERFs members identified through transcriptome analysis. Evolutionary relationship analysis showed that the 17 PpERFs could be further divided into three clades: I, II, and III, from which Clade I comprised the most significant proportion (Fig. 3 A). The physicochemical properties of the 17 PpERF proteins were variable. For instance, the number of amino acids ranged from 149 aa to 327 aa, the MW varied from 16.76 kDa to 36.74 kDa, and the pI ranged from 4.75 to 9.51. According to the subcellular localization prediction, the 17 PpERFs were mainly located in the nucleus and cytoplasm. It is well established that members of the ERF subfamily could interact with other proteins to regulate the expression of downstream target genes related to hormone signal transduction [32]. Our interaction prediction results showed that the 17 PpERF proteins could interact with various transcription factors or proteins (Table 1). The sand pear (*Pyrus pyrifolia*) genome contains 17 chromosomes [30]. The chromosomal distribution analysis indicated that the17 *PpERF* genes were randomly distributed across 11 chromosomes, mainly on both ends of the chromosome (Fig. 2). Similar chromosomal distribution of *AP2/ERF* genes was reported in cultivated peanut [33].

Moreover, motif composition and domain analysis showed that the protein-coding sequences of the 17 PpERFs had variable motifs and conserved domains (Fig. 3 B, C). We identified five motifs across all PpERFs. Interestingly, all members contained motif 1, while most PpERFs had motif 2. These two motifs are the conserved AP2 domain. Only *PpERF027* and *PpERF025-L* grouped into clade III had motif 5, which may provide a particular function. All 17 AP2/ERF family members have a conserved AP2 domain.

Furthermore, we observed the same exon-intron structures in the 17 *PpERF* genes, with most *PpERFs* having no exon. Similar results have been reported in white pears [20]. The lack of exon might be related to the sensitivity of gene transcription regulation [34]. Cis-elements analysis showed that the 17 *PpERF* genes had various hormone-responsive elements in their promoter. These cis-elements could play a crucial role in regulating the expression of *PpERF* genes during fruit development and ripening.

*AP2/ERF* genes showed distinct expressions at different stages of fruit development. In this study, cluster analysis showed that the expression levels of *PpERF061* and *PpERF113* were higher at 180 DAH. *PpERF5-L,* and *PpERF017* exhibited higher expression levels at the early stage of fruit development. Besides, *PpERF51B-L* was mainly expressed during fruit ripening. These results suggest that *PpERF061* and *PpERF113* may play an important role in fruit senescence, while *PpERF5-L* and *PpERF017* could be involved in early fruit development. Whereas *PpERF51B-L* could mainly regulate fruit ripening.

## Conclusion

This study demonstrates the identification of the *AP2/ERF* gene family in sand pear through combined transcriptome and genome-wide analyses. A total of 234 unique *PpERF* genes were identified. We further analyzed the evolutionary relationship, physicochemical properties, chromosomal distributions, conserved motifs, domains, gene structures, cis-acting elements, and expression patterns in different tissues and fruit development stages for the 17 *PpERF* genes identified through transcriptome analysis. Additionally, expression analysis showed that five genes, including *PpERF061*, *PpERF113*, *PpERF51L-B*, *PpERF5-L,* and *PpERF017,* could be involved in regulating sand pear fruit development, ripening, and senescence. These results provide candidate genes to study the function of *PpERFs* in regulating fruit quality and prolonging the shelf life of sand pear. Overall, this study provides crucial information to study the role of *AP2/ERF* genes during fruit ripening and senescence in sand pear.

## Materials and methods

### Plant materials

Sand pear (*Pyrus pyrifolia* Nakai. ‘Whangkeumbae’) fruits were collected at 30, 60, 90, 120, 130, 140, 145, and 150 days after full bloom (DAFB) from the experimental farm of Hebei Agricultural University, China. Naturally ripened fruits at 150 DAFB were placed at room temperature for 5, 10, 15, 20, 25, and 30 d. The samples were ground into a powder with liquid nitrogen for RNA isolation [35].

### Identification of AP2/ERF gene family members in sand pear

In our previous study, 17 proteins encoding the AP2 domain were identified through transcriptome analysis of ‘Whangkeumbae’ (*Pyrus pyrifolia*) [29]. In this study, the sand pear (*Pyrus pyrifolia*) cultivar ‘Cuiguan’ genome file (assembly number: GWHBAOS00000000) [30] was retrieved from the NGDC (https://ngdc.cncb.ac.cn/). The *Arabidopsis thaliana* AP2/ERF protein sequences were downloaded from the NCBI database (https://www.ncbi.nlm.nih.gov/). Then, two approaches were employed to identify the AP2/ERF gene family members in *Pyrus pyrifolia*. First, the HMM of the AP2 domain (PF00847) was downloaded from the Pfam database (http://pfam.xfam.org/) and used to align with all *Pyrus pyrifolia* genome protein sequences to retrieve *AP2/ERF* genes using the software SPDE [36], TBtools [37] and GFAP [38]. Moreover, the protein sequences of *Arabidopsis thaliana* AP2/ERF members were used to perform an extensive local BLASTP search with a threshold E-value of <1e-5 against *Pyrus pyrifolia* genome sequence to obtain candidate PpERFs. Redundant sequences were removed from the above results. The conserved domains of the candidate *PpERF* genes were searched for batch comparison to verify whether they contained AP2 conserved domains using the Pfam database and the NCBI-CDD (https://www.ncbi.nlm.nih.gov/Structure/cdd/wrpsb.cgi).

The sand pear AP2/ERF protein sequences identified from the transcriptome and genome analyses were integrated for evolutionary analysis. Multiple sequence alignment analysis of AP2/ERF proteins was performed using the ClustalW method. The phylogenetic tree was constructed using the NJ method by MEGA7.0 [39] with 1000 bootstrap replicates. The online tool ChiPlot (https://www.chiplot.online/#) was further used to improve the phylogenetic tree.

### Phylogenetics, conserved motifs, domains, and gene structures analyses of the 17 PpERFs

The evolutionary relationship of the 17 PpERFs identified by transcriptome sequencing was analyzed by MEGA 7.0 software. The proteins sequences of the 17 PpERFs were submitted to the NCBI-CDD and online MEME tool (https://meme-suite.org/meme/index.html) to identify domains and motifs at E-value (0.01) and E-value (0.05), respectively. The *Pyrus pyrifolia* cultivar ‘Cuiguan’ genome annotation file was obtained from the NGDC for gene structure analysis. The gene structures were analyzed using the Gene Structure Display Server (GSDS) tool (http://gsds.cbi.pku.edu.cn/) based on the alignments of CDS sequences with their corresponding genomic DNA sequences.

The phylogenetic tree, conserved motifs, domains, and gene structure diagrams of the 17 PpERFs, were constructed using the TBtools software. For chromosomal localization prediction, the annotation file of *Pyrus pyrifolia* genome was retrieved from the NGDC and analyzed using the local TBtools. The physicochemical properties, including protein size, MW, and PI, were predicted by the online tool Expasy ProtParam (https://web.expasy.org/protparam/). Protein-protein interaction predictions were carried out using the STRING software (https://string-db.org/). The subcellular localization of the 17 PpERF members was investigated using the Plant-mPLoc online tool (http://www.csbio.sjtu.edu.cn/bioinf/plant-multi/).

**Cis-acting elements analysis of the promoters of 17** ***PpERF*** **genes.**

The 17 PpERF protein sequences were BLASTP searched against the *Pyrus pyrifolia* ‘Cuiguan’ genome using the online Genome Database for Rosaceae (GDR) blast tools (https://www.rosaceae.org). The putative 2000 bp promoter regions were extracted using the TBtools software. Then, cis-regulatory elements were identified using the PlantCARE database (http://bioinformatics.psb.ugent.be/webtools/plantcare/html/). Finally, we chose hormonal-responsive cis-elements related to ripening and senescence. The TBtools and Adobe Illustrator were used to visualize the location and number of cis-elements.

### RNA extraction and quantitative RT-PCR analysis

Total RNA was extracted from various tissues of sand pear at different developmental stages, using an RNAprep Pure Plant Plus Kit (Tian Gen, Beijing, China) according to the instructions. cDNA was synthesized with a FastQuant RT Kit (with gDNase) (Tian Gen, Beijing, China) following the manufacturer’s instructions. The expression levels of the 17 *PpERF* genes were analyzed using qRT-PCR via a Magic SYBR mixture according to the manufacturer’s instructions (CoWin Biosciences, China) in the detection system (Mastercycler ep realplex 4, Eppendorf AG, Hamburg, Germany).

The expressions of the 17 *PpERF* genes were investigated in different tissues, including shoots, stems, leaves, petals, anthers, and mesocarp. Based on their expression in the mesocarp, *PpERF5-L*, *PpERF017*, *PpERF51B-L*, *PpERF061,* and *PpERF113* were identified as candidate genes related to fruit, development, ripening, and senescence for further analysis. The expression levels of these five *PpERF* genes were determined during fruit development and storage. This experiment was performed with three repeats. All the primers used for the qRT-PCR experiment are listed in Additional file 3: Table S2.

### Statistical analysis

The relative expression values of *PpERFs* were analyzed using SPSS with the Duncan test. GraphPad Prism 9.0.0 software was used to draw charts. The data are displayed as the mean ± S.D. (*n* = 3).

## Supplementary Information

**Additional file 1.**

**Additional file 2.**

**Additional file 3.**

## Data Availability

The genome sequence information contained in this study were obtained from following websites. *Pyrus pyrifolia* cultivar ‘Cuiguan’ genome file from the National Genomics Date Center (NGDC): https://ngdc.cncb.ac.cn/. Arabidopsis protein sequences from the National Center for Biotechnology Information (NCBI) database: https://www.ncbi.nlm.nih.gov/. The data sets supporting the conclusions of this study are included within the article and its additional files.

## Abbreviations

ERF: Ethylene response factor
ACC: 1-aminocypropane-1-carboxylic acid
ACS: 1-aminocypropane-1-carboxylic synthase
SAM: S-adenosine methionine
ACO: ACC oxidase
EIN3: ethylene insensitive 3
EIN3/EIL: EIN3/LIKE
TFs: transcription factors
AP2: APETALA2
aa: amino acid;
PHD: plant homeodomain
MeJA: Methyl Jasmonate
C4HC3: Cys 4-His-Cys 3
DEGs: differentially expressed genes
DAFB: days after full bloom
DAH: days after harvest
NGDC: National Genomics Date Center
NCBI: National Center for Biotechnology Information
HMM: Hidden Markov Model
Chr: Chromosome
MW: molecular weight
kDa: kilo Dalton
pI: isoelectric point
E-value: Expected value
CDD: Conserved Domain Database
NJ: Neighbor-Joining
MEME: Motif Elicitation
GSDS: Gene Structure Display Server
GDR: Genome Database for Rosaceae.

## References

[1] Zhang HY, Zhao L, Fan CX (2020). Impact of methyl salicylate on storage quality, ethylene action, and protein profiling of ‘Zaosu’ pear (*Pyrus bretschneideri*). Sci Hortic.

[2] Yue PT, Lu Q, Liu Z (2020). Auxin-activated MdARF5 induces the expression of ethylene biosynthetic genes to initiate apple fruit ripening. New Phytol.

[3] Adams DO, Yang SF (1979). Ethylene biosynthesis: identification of 1-aminocyclopropane-1-carboxylic acid as an intermediate in the conversion of methionine to ethylene. Proc. Natl. Acad. Sci. U S A.

[4] Wang KL, Li H, Ecker JR (2002). Ethylene biosynthesis and signaling networks. Plant Cell.

[5] Rashid M, He GY, Yang GX (2012). AP2/ERF transcription factor in rice: genome-wide canvas and syntenic relationships between monocots and eudicots. Evol Bioinformatics Online.

[6] Liu MC, Gomes BL, Mila I (2016). Comprehensive profiling of ethylene response factor expression identifies ripening-associated ERF genes and their link to key regulators of fruit ripening in tomato. Plant Physiol.

[7] Hu YN, Han ZY, Sun YQ (2020). ERF4 affects fruit firmness through TPL4 by reducing ethylene production. Plant J.

[8] Xu ZS, Chen M, Li LC (2011). Functions and application of the AP2/ERF transcription factor family in crop improvement. J Integr Plant Biol.

[9] Reboledo G, Agorio A, Vignale L (2022). The moss-specific transcription factor PpERF24 positively modulates immunity against fungal pathogens in *Physcomitrium patens*. Front Plant Sci.

[10] Debbarma J, Sarki YN, Saikia B (2019). Ethylene response factor (ERF) family proteins in abiotic stresses and CRISPR-Cas9 genome editing of ERFs for multiple abiotic stress tolerance in crop plants: a review. Mol Biotechnol.

[11] Li YC, Zhu BZ, Xu WT (2007). LeERF1 positively modulated ethylene triple response on etiolated seedling, plant development and fruit ripening and softening in tomato. Plant Cell Rep.

[12] Deng H, Chen Y, Liu ZY (2022). SlERF.F12 modulates the transition to ripening in tomato fruit by recruiting the co-repressor TOPLESS and histone deacetylases to repress key ripening genes. Plant Cell.

[13] Hu YN, Han ZY, Wang T (2022). Ethylene response factor MdERF4 and histone deacetylase MdHDA19 suppress apple fruit ripening through histone deacetylation of ripening-related genes. Plant Physiol.

[14] Wu T, Liu HT, Zhao GP (2020). Jasmonate and ethylene-regulated ethylene response factor 22 promotes lanolin-induced anthocyanin biosynthesis in 'Zaosu' pear (*Pyrusbretschneideri* Rehd.) fruit. Biomolecules.

[15] Wang XB, Zeng WF, Ding YF (2019). Peach ethylene response factor PpeERF2 represses the expression of ABA biosynthesis and cell wall degradation genes during fruit ripening. Plant Sci.

[16] Wang XB, Zeng WF, Ding YF (2019). *PpERF3* positively regulates ABA biosynthesis by activating *PpNCED2/3* transcription during fruit ripening in peach. Hortic Res.

[17] Cai HF, Han S, Wang H (2021). The regulation of 1-methylcyclopropene treatment on the subfamily genes expression of ethylene response factors in peaches during storage. Acta. Sci. Pol. Technol. Aliment.

[18] Girardi CL, Rombaldi CV, Cero JD (2013). Genome-wide analysis of the AP2/ERF superfamily in apple and transcriptional evidence of ERF involvement in scab pathogenesis. Sci. Hortic.

[19] Riechmann JL, Heard J, Martin G (2000). Arabidopsis transcription factors: genome-wide comparative analysis among eukaryotes. Science.

[20] Li XL, Tao ST, Wei SW (2018). The mining and evolutionary investigation of AP2/ERF genes in pear (*Pyrus*). BMC Plant Biol.

[21] Xie XL, Shen SL, Yin XR (2014). Isolation, classification and transcription profiles of the AP2/ERF transcription factor superfamily in citrus. Mol Biol Rep.

[22] Mizuno S, Hirasawa Y, Sonoda M (2006). Isolation and characterization of three DREB/ERF-type transcription factors from melon (*Cucumis melo*). Plant Sci.

[23] Zhang CH, Shangguan LF, Ma RJ (2012). Genome-wide analysis of the AP2/ERF superfamily in peach (*Prunus persica*). Genet Mol Res.

[24] Licausi F, Giorgi FM, Zenoni S (2010). Genomic and transcriptomic analysis of the AP2/ERF superfamily in *Vitis vinifera*. BMC Genomics.

[25] Jiang Q, Wang Z, Hu GM (2022). Genome-wide identification and characterization of *AP2/ERF* gene superfamily during flower development in *Actinidia eriantha*. BMC Genomics.

[26] Sakuma Y, Liu Q, Dubouzet JG (2002). DNA-binding specificity of the ERF/AP2 domain of Arabidopsis DREBs, transcription factors involved in dehydration-and cold-inducible gene expression. Biochem Biophys Res Commun.

[27] Zhang GY, Chen M, Chen XP (2008). Phylogeny, gene structures, and expression patterns of the *ERF* gene family in soybean (*Glycine max* L.). J Exp Bot.

[28] Xing HT, Jiang YS, Zou Y (2021). Genome-wide investigation of the *AP2/ERF* gene family in ginger: evolution and expression profiling during development and abiotic stresses. BMC Plant Biol.

[29] Shi HY, Cao LW, Xu Y (2021). Transcriptional profiles underlying the effects of salicylic acid on fruit ripening and senescence in pear (*Pyrus pyrifolia* Nakai). J Integr Agr.

[30] Gao YH, Yang QS, Yan XH (2021). High-quality genome assembly of 'Cuiguan' pear (*Pyrus pyrifolia*) as a reference genome for identifying regulatory genes and epigenetic modifications responsible for bud dormancy. Hortic. Res.

[31] Ohme-Takagi M, Shinshi H (1995). Ethylene-inducible DNA binding proteins that interact with an ethylene-responsive element. Plant Cell.

[32] Chen HY, Hsieh EJ, Cheng MC (2016). ORA47 (octadecanoid-responsive AP2/ERF-domain transcription factor 47) regulates jasmonic acid and abscisic acid biosynthesis and signaling through binding to a novel cis-element. New Phytol.

[33] Cui YY, Bian JX, Guan Y (2022). Genome-wide analysis and expression profiles of ethylene signal genes and Apetala2/ethylene-responsive factors in Peanut (*Arachis hypogaea* L.). front. Plant Sci.

[34] Song ZP, Pan FL, Yang C (2019). Genome-wide identification and expression analysis of *HSP90* gene family in *Nicotiana tabacum*. BMC Genet.

[35] Shi HY, Zhang YX, Chen L (2019). Expression and regulation of *PpEIN3b* during fruit ripening and senescence via integrating SA, glucose, and ACC signaling in pear (*Pyrus pyrifolia* Nakai. Whangkeumbae). Genes.

[36] Xu D, Lu ZC, Jin KM (2021). SPDE: a multi-functional software for sequence processing and data extraction. Bioinformatics.

[37] Chen CJ, Chen H, Zhang Y (2020). TBtools - an integrative toolkit developed for interactive analyses of big biological data. Mol Plant.

[38] Xu D, Jin K, Jiang H, et al. GFAP: ultra-fast and accurate gene functional annotation software for plants. bioRxiv. 2022. 10.1101/2022.01.05.475154.

[39] Kumar S, Stecher G, Tamura K (2016). MEGA7: molecular evolutionary genetics analysis version 7.0 for bigger datasets. Mol. Biol. Evol.

