# Supplementary figures and images for: Genome-wide identification and molecular characterization of the AP2/ERF superfamily members in sand pear (Pyrus pyrifolia)
Source: BMC Genomics. 2023 Jan 19;24:32. doi: 10.1186/s12864-022-09104-4 (PMC9854111; doi:10.1186/s12864-022-09104-4)

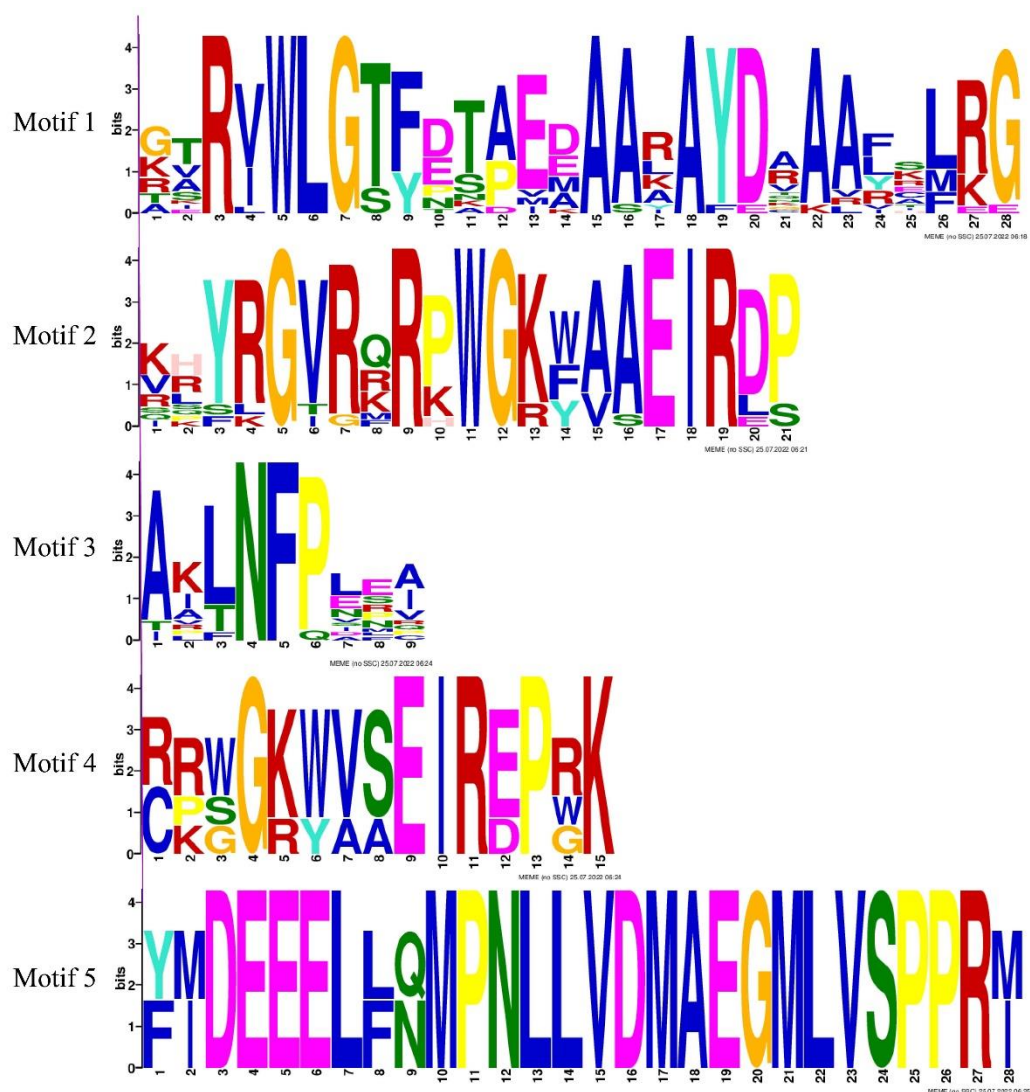

**Fig. S1** Protein sequences of conserved motifs identified in 17 PpERFs.

Supplement: Supplementary file 1 — Additional file 1. [file 12864_2022_9104_MOESM1_ESM.pdf]
