# Supplementary material for: Genome-wide identification and molecular characterization of the AP2/ERF superfamily members in sand pear (Pyrus pyrifolia)
Source: BMC Genomics. 2023 Jan 19;24:32. doi: 10.1186/s12864-022-09104-4 (PMC9854111; doi:10.1186/s12864-022-09104-4)
Supplement: Supplementary file 2 — Additional file 2. [file 12864_2022_9104_MOESM2_ESM.pdf]

Table S1. Subfamilies and numbers of *AP2/ERF* genes in different species.

Subfamily	*Pyrus pyrifolia*	*Pyrus bretschneideri*	*Malus domestica*	*Prunus persica*	*Vitis vinifera*	*Actinidia eriantha*	*Arabidopsis thaliana*	*Glycine max*	*Oryza sativa*	*Zingiber officinale*
AP2	38	26	51	21	20	34	18	26	29	35
ERF	188	155	195	104	122	119	122	120	145	120
RAV	8	9	6	5	6	3	6	2	5	3
Soloist	0	1	7	1	1	2	1	0	1	5
Total	234	191	259	131	149	158	147	148	180	163
