# Supplementary material for: Genome-wide identification and molecular characterization of the AP2/ERF superfamily members in sand pear (Pyrus pyrifolia)
Source: BMC Genomics. 2023 Jan 19;24:32. doi: 10.1186/s12864-022-09104-4 (PMC9854111; doi:10.1186/s12864-022-09104-4)
Supplement: Supplementary file 3 — Additional file 3. [file 12864_2022_9104_MOESM3_ESM.pdf]

Table S2. Primers for quantitative RT-PCR (qRT-PCR).

Gene name	Forward primer	Reverse primer
PpERF51B-L	TGCGGCGGTTCTCAATTTTCCAGTT	CAGTTCTTCCAAGTAATCCGTTCCC
PpERF12	CATCGACCTCAACGCTCCCTCC	AAAAGACGCCGTACTGCCGAAAC
PpERF109-L	GGTCATGGAGAAGGAGGAGGAGAA	GTTCGTGGATAACCATGCCATTAAG
PpERF027	AATGCAGAAGCACGAGGGTGATGA	AATAGCTCCACAGACGGCCTTCTC
PpERF061	GCGACAGTTCTAGACTGAACGCCG	TCGTGGCCAAAACCGTCGCCAAAG
PpERF118-L	GATGAGACATTGGGCCCTCTTCAT	AAAGTCACAGTCGGGTAGACTGCT
PpERF025-L	GATATGCTGGCTCCTCAGGAGAGG	TTCTCCGTCGGAATTCCCAGGGG
PpERF14-L	CAACAGAGAGACGAGGCGAAGGA	TACGACCATTACTTGTGGATAACC
PpERF5-L	GTCTTCGAGGCCCTTCGGCCCG	AGTTTTCTGGGTCTGGGTACTCA
PpERF3a	TTCAATCGACGACGGTCGCTGCTG	GACTCCCCATCCACATCCAACGGT
PpERF1a	CGGTTCAGCAGTCGGCTCAAGCCT	TTCAACAAAGCCCTTGAGCCGCGC
PpERF113	CCGTATTGTTAATCCTCAGACAGC	CGATGGTTGTGAATCATGTGATGC
PpERF022-L	AATGGAGGCATTTCCAACGGAGGA	ATGTCCCACAAGTCTCCCCCTAAC
PpERF3	ATACGACGCTGCCGCTCGTTCC	AGGGTCGGATGTGGGCCTGGGC
PpERF017	GTCCCTGTCGGAGGGCAGTGGGA	GCGTAGTAGTCAACGGCCGGCGTT
PpERF2	CTCACTTCAAAATGTCTACACATG	TTGACTGGTTGGAACTCGAAATAT
PpERF098	CGTGACTCAAGTAGGCATGGCGTG	CAACCACGAGTTCGCCCCTGCATA
PpUBI	TGCAGATCTTCGTGAAAACCCTAAC	CATAGCACTTGCGGCAGATCATCT
